# Human-macaque comparisons illuminate variation in neutral substitution rates

**DOI:** 10.1186/gb-2008-9-4-r76

**Published:** 2008-04-30

**Authors:** Svitlana Tyekucheva, Kateryna D Makova, John E Karro, Ross C Hardison, Webb Miller, Francesca Chiaromonte

**Affiliations:** 1 Center for Comparative Genomics and Bioinformatics, The Pennsylvania State University, University Park, PA 16802, USA; 2 Department of Statistics, The Pennsylvania State University, University Park, PA 16802, USA; 3 Department of Biology, The Pennsylvania State University, University Park, PA 16802, USA; 4 Department of Computer Science and System Analysis, Miami University, Oxford, OH 45056, USA; 5 Department of Microbiology, Miami University, Oxford, OH 45056, USA; 6 Department of Biochemistry and Molecular Biology, The Pennsylvania State University, University Park, PA 16802, USA; 7 Department of Computer Science and Engineering, The Pennsylvania State University, University Park, PA 16802, USA

## Abstract

**Background:**

The evolutionary distance between human and macaque is particularly attractive for investigating local variation in neutral substitution rates, because substitutions can be inferred more reliably than in comparisons with rodents and are less influenced by the effects of current and ancient diversity than in comparisons with closer primates. Here we investigate the human-macaque neutral substitution rate as a function of a number of genomic parameters.

**Results:**

Using regression analyses we find that male mutation bias, male (but not female) recombination rate, distance to telomeres and substitution rates computed from orthologous regions in mouse-rat and dog-cow comparisons are prominent predictors of the neutral rate. Additionally, we demonstrate that the previously observed biphasic relationship between neutral rate and GC content can be accounted for by properly combining rates at CpG and non-CpG sites. Finally, we find the neutral rate to be negatively correlated with the densities of several classes of computationally predicted functional elements, and less so with the densities of certain classes of experimentally verified functional elements.

**Conclusion:**

Our results suggest that while female recombination may be mainly responsible for driving evolution in GC content, male recombination may be mutagenic, and that other mutagenic mechanisms acting near telomeres, and mechanisms whose effects are shared across mammalian genomes, play significant roles. We also have evidence that the nonlinear increase in rates at high GC levels may be largely due to hyper-mutability of CpG dinucleotides. Finally, our results suggest that the performance of conservation-based prediction methods can be improved by accounting for neutral rates.

## Abstract 2

The evolutionary distance between human and macaque is particularly attractive for investigating neutral substitution rates, which were calculated as a function of a number of genomic parameters.

## Background

A better understanding of mutation processes is important for investigating the causes of human genetic diseases and studying the dynamics of molecular evolution. Additionally, identifying and quantifying the effects of genomic parameters that predict neutral substitution rates is crucial for pursuing a more realistic modeling of neutral versus selective processes acting on the human genome. Improvements in these models may play a role in the development of more accurate computational methods for the identification of functional elements.

Rates of nucleotide substitution (divergence) at neutral sites are known to vary within mammalian and other genomes [1-4]. Moreover, such rates have been shown to co-vary with other measures of change in chromosomal DNA, including rates of small insertions and deletions, insertions of transposable elements, and single nucleotide polymorphisms (SNPs) [3,5-7], leading to the hypothesis that some regions in the genome are more prone to evolutionary change of any kind compared with other regions [3].

Interestingly, neutral substitution rates have also been shown to correlate with GC content, local recombination rates, and distance to telomeres [3,8]. The relationship between divergence and GC content was found to be biphasic, that is, to show a curved trend [3], perhaps reflecting the presence of mutational hotspots at CpG sites [8]. Recombination rate is another important predictor of mammalian divergence, and mechanistically can lead to increased mutation rates through incorrect repair of double-strand breaks [9], although for humans this has not been demonstrated unequivocally and is still debated [10].

Another area of interest is the scale and evolutionary conservation of variation in substitution rates. Many studies have indicated that either whole autosomes [2] or regions of conserved synteny [11] are 'units' within which substitution rates are relatively homogeneous. However, a recent study indicated that regional variation in divergence, at least in rodents, is better captured by segments approximately 1 Mb in size, and that variation within autosomes is more significant than that among autosomes [12]. Sex chromosomes appear to be outliers in terms of genomic divergence, primarily because they spend different relative amounts of time in the male and female germlines compared to autosomes [13].

While a complete understanding of all biological mechanisms leading to variation in neutral substitution rates across the genome remains elusive, it is plausible that at least some of these mechanisms are conserved over relatively long evolutionary distances. For instance, both mouse-specific and rat-specific substitution rates are positively correlated with rodent-primate substitution rates [14], suggesting shared mechanisms persisting over approximately 90 million years [15]. Additionally, a positive correlation exists in substitution rates of homologous X- and Y-chromosomal introns that diverged from each other approximately 100 million years ago [16].

Relative to previous studies that concentrated on human-mouse [3], mouse-rat [12] or human-chimpanzee [8] comparisons, the availability of the macaque genome provides an appealing evolutionary distance to investigate regional variation in the human lineage for the following reasons. First, the human-macaque divergence is smaller than that for human-mouse, and thus can be estimated more accurately. Second, the human-macaque divergence is greater than that for human-chimpanzee, and thus expected to be less affected by biases due to ancestral polymorphism [13].

In this study, we employ multiple regression analysis to investigate regional variation in human-macaque divergence as a function of several genomic features, performing separate analyses for neutral substitution rates computed on all sites, non-CpG sites and CpG sites, and using ancestral repeats as a model for neutral DNA [3]. In addition to our regressions, separating CpG and non-CpG sites allows us to shed some light on the biphasic relationship between divergence and GC content observed in several studies (for example, [3]). Utilizing our data and some theoretical derivations, we show that increased substitution rates at high GC levels can be explained as an effect of the hypermutability of CpG dinucleotides. Finally, because of the significant consequences that regional variation in divergence may have on algorithms for the identification of putative functional elements, we investigate the association between human-macaque neutral substitution rates and both computationally predicted and experimentally validated functional elements.

## Results and discussion

### Explaining neutral rates using multiple regression analysis

We start with results from the regressions of human-macaque neutral substitution rates computed from non-CpG sites and all sites on various candidate predictors. Both rates are computed on alignments of selected classes of interspersed repetitive elements (ancestral repeats) in 1 Mb non-overlapping windows of the human genome covering autosomes and chromosome X. In the set of repeats employed for our analyses, less than 2% of the bases belonged to highly conserved elements as assessed by phyloHMM [17]; therefore, we do not expect sizeable biases due to the inclusion of potentially functional sequences. We estimated substitution rates using both Jukes-Cantor (JC) [18] and Hasegawa-Kishino-Yano (HKY) [19] substitution models. The JC model has a single free parameter and can reliably estimate rates from fewer sites. The more complex HKY model has four free parameters, accounting for differences in transition versus transversion rates and equilibrium frequencies of the four nucleotides (the HKY model may thus be more appropriate for computing substitution rates at CpG sites; see below). The two models showed good agreement, with correlations between estimated rates as high as 0.99 for all and non-CpG sites, and 0.94 for CpG sites, and very similar regression results. Throughout the paper we report results obtained using the simpler JC model (regression output for HKY model rates is provided in Additional data file 1).

Excluding windows located in segmental duplications or not having a sufficient number of informative ancestral repeat bases (see Materials and methods) resulted in a set of 2,270 windows. For each window, we computed human GC content and obtained exon density, SNP density, and recombination rates (both male and female) from annotations at the UCSC Human Genome Browser [20]. To derive the distance to a telomere for a given window, we computed: the average distance between the centers of human repeats considered in the window and the closest human telomere; and the average distance between the centers of orthologous macaque repeats and the closest macaque telomere, and took the minimum between these two averages. This provides a predictor that accounts for proximity to telomeres on both the human and macaque sides, and is thus able to explain elevated mutation rates in non-telomeric human regions having macaque orthologs close to telomeres (for example, on human chromosome 2, where two arms correspond to different macaque chromosomes [21], and on human chromosome 3, where rearrangements between human and macaque occurred [22]). More details on data preparation are provided in the Materials and methods section.

**Figure 1:**
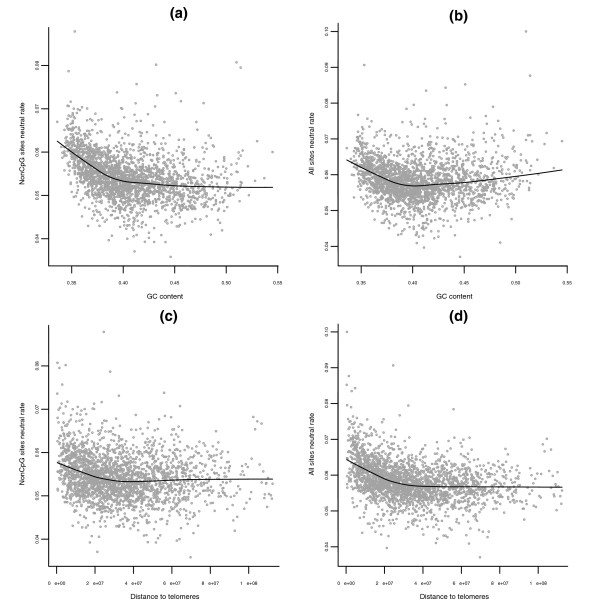
Neutral rates, GC and distance to telomeres. **(a-d) **Scatter plots of human-macaque neutral substitution rates from non-CpG and all sites in ancestral repeats against human GC content ((a) and (b), respectively) and distance to telomeres ((c) and (d), respectively). Each point represents one of 2,270 windows of size 1 Mb. Lowess smoothers are superimposed to the plots to help visualize the relationships. These non-parametric fits reveal some curvature in the way GC content and distance to telomeres are related to neutral substitutions, which is consistent with the significant quadratic terms in our regression fits.

The results of our regressions for neutral rates at non-CpG sites and all sites (Table 1) confirm important roles for previously studied predictors [3,23-25]. In both regressions, GC content is the strongest predictor, explaining 12% and 14% of the variability for non-CpG and all sites, respectively. The significant negative linear coefficients and large, highly significant positive quadratic coefficients confirm a curved association (see also scatter plots in Figure 1a,b). In addition, in both regressions, exons and SNPs are significant predictors, with negative and positive signs, respectively.

**Table 1:** Regression results for neutral substitution rates estimated from non-CpG and all sites

	Non-CpG sites				All sites			
		
Predictors	*t *value*	Significance^†^	VIF^‡^	Variability explained^§^	*t *value*	Significance^†^	VIF^‡^	Variability explained^§^
X chromosome/autosome indicator	13.94	<10^-4^	1.2	0.08	15.25	<10^-4^	1.3	0.09
GC content								
Linear term	-10.34	<10^-4^	3.7	0.12	-5.08	<10^-4^	3.4	0.14
Quadratic term	15.85	<10^-4^	1.3		18.78	<10^-4^	1.2	
Exon density	-7.03	<10^-4^	2.4	0.02	-9.37	<10^-4^	2.4	0.03
SNP density	6.25	<10^-4^	1.2	0.02	6.85	<10^-4^	1.2	0.02
Male recombination rate	3.69	0.003	1.6	0.01	4.46	<10^-4^	1.6	0.01
Female recombination rate	NS	NS	NS	NS	NS	NS	NS	NS
Distance to telomere								
Linear term	-12.33	<10^-4^	2.5	0.06	-16.78	<10^-4^	2.5	0.11
Quadratic term	7.63	<10^-4^	2.0		10.77	<10^-4^	2.0	
Mouse-rat orthologous neutral rate	7.95	<10^-4^	1.8	0.09	6.64	<10^-4^	1.4	0.07
Dog-cow orthologous neutral rate	10.56	<10^-4^	1.3		10.41	<10^-4^	1.4	
Multiple R^2^				0.52				0.53
Adjusted R^2^				0.52				0.52

Non-CpG and all sites were taken in ancestral repeats orthologous to mouse, rat, dog and cow for each of 2,270 windows of size 1 Mb. *t value, test statistic of null hypothesis that each predictor's coefficient is equal to zero; ^†^*p*-values adjusted for multiple tests (using Bonferroni correction); ^‡^VIF, variance inflation factor; ^§^relative contribution to explained variability computed for each predictor. NS, non-significant

In an attempt to elucidate the role of male- and female-specific recombination, we consider sex-specific recombination rates (instead of sex-averaged ones). In both regressions, male recombination is a significant positive predictor, while female recombination is not significant. This suggests that sex-averages tend to obscure the role of recombination; once male recombination is considered as a separate predictor, its significance emerges, providing evidence for a possible mutagenic effect (also reported in [2,8]). Moreover, our results are consistent with Meunier and Duret's hypothesis that female recombination acts mostly through an increase in GC content [24]; since GC content is included as a predictor in our regressions, female recombination becomes non-significant. Interestingly, a new study of biased clustered substitutions revealed similar patterns [26]. Another factor at play may be that female recombination rates change faster than their male counterparts over evolutionary time [27], and this may dilute the observable association between female recombination and neutral substitution rates.

The depletion of substitutions on chromosome X relative to autosomes has been noted in previous studies (for example, [28,29]). The autosomes/X indicator (see Materials and methods) is a prominent positive predictor in both regressions (explaining 8% and 9% of the variability for non-CpG and all sites, respectively). Thus, all other predictors being equal, autosomal windows tend to have substantially higher substitution rates than X windows. This confirms the important role of male mutation bias [13,30], and suggests a replication-dependent origin for the observed divergence [29]. However, it must be noted that recombination could also be related to the depletion of substitutions on chromosome X. Indeed, even though average recombination rates are about equal between autosomes and chromosome X [31], evolutionary recombination rates (that is, rates adjusted for spending less time in the recombining sex, a female) are, in fact, two-thirds lower for the latter.

Distance to telomeres emerges as another important predictor in both regressions (explaining 6% and 11% of the variability for non-CpG and all sites, respectively), and the relationship between substitution rates and distance to telomeres appears to be curved, with highly significant linear (negative) and quadratic (positive) coefficients (Figure 1c,d). Recombination rates, in particular male-specific ones, correlate with distance to telomeres [23]. However, since human recombination rates are included in our regressions, the prominent role of distance to telomeres is not a reflection of this correlation. Because distance to telomeres is defined to account for proximity to telomeres also in macaque, it could at least partially capture the effects of macaque recombination - whose rates may well differ from human (recombination rates differ between human and chimpanzee [32], as well as among human, mouse, and rat [31]). Unambiguously separating recombination from other telomeric effects would require data on recombination rates in macaque that are currently unavailable. Nevertheless, given the strength of distance to telomeres as a predictor, our results suggest the existence of additional mutagenic mechanisms that increase neutral substitution rates in subtelomeric regions. Increased divergence near telomeres has been linked to direct and indirect effects of large-scale chromosomal structure, and other lineage-specific factors [33]. Additionally, the recombination rates used in our study (from [23]) represent crossover rates; it is known that the proportion of recombination events actually resulting in crossovers varies across the genome [24] and might be peculiar near telomeres. Interestingly, rates of small insertions estimated using human-chimpanzee alignments are also elevated near telomeres [7].

Finally, we calculate neutral substitution rates in orthologous regions from mouse-rat and dog-cow alignments. Correlations between orthologous neutral rates computed at all sites tend to be lower than those between rates computed at non-CpG sites (Table 2), perhaps because CpG sites diverge rapidly and independently in separate species due to their hypermutability. Using orthologous neutral rates as predictors in our regressions is a way to assess the presence of other mechanisms affecting human-macaque substitution rates, as long as the orthologies are reliable and the mechanisms are 'conserved', that is, their effects are shared, across the mammalian species under consideration. For both regressions, orthologous substitution rates are remarkably strong positive predictors, explaining 9% and 7% of the variability for non-CpG and all sites, respectively. These percentages are comparable to those explained by the autosome/X indicator and distance to telomeres.

**Table 2:** Correlations between neutral substitution rates in orthologous regions

	All sites			Non-CpG sites		
		
	Human-macaque	Mouse-rat	Dog-cow	Human-macaque	Mouse-rat	Dog-cow
All sites						
Human-macaque		0.28	0.42	0.9	0.28	0.48
Mouse-rat	<10^-4^		0.05	0.37	0.89	0.22
Dog-cow	<10^-4^	0.02		0.27	-0.13	0.87
						
Non-CpG sites						
Human-macaque	<10^-4^	<10^-4^	<10^-4^		0.44	0.45
Mouse-rat	<10^-4^	<10^-4^	0.51	<10^-4^		0.26
Dog-cow	<10^-4^	<10^-4^	<10^-4^	<10^-4^	<10^-4^	

Upper-right off-diagonal: pair-wise Pearson's correlation coefficients between human-macaque, mouse-rat and dog-cow orthologous substitution rates estimated from non-CpG and all sites in ancestral repeats orthologous to mouse, rat, dog and cow for each of 2,270 windows of size 1 Mb. Lower-left off diagonal: *p*-values expressing significance of the correlation coefficients.

The overall percentage of variability explained (R^2^) is approximately 52% in both regressions, which is among the highest reported in this type of study. Moreover, the regressions are satisfactory in terms of statistical diagnostics; residuals show neither significant trends unaccounted for by the regression equations nor strong departures from a Gaussian distribution, justifying the use of standard *t*-tests for regression coefficients.

To study substitution rates at CpG sites in ancestral repeats, we recalculated human-macaque neutral substitution rates using the same set of windows and the same repeat families, but relaxing the requirement that repeats align also with dog, cow, mouse, and rat. This requirement was imposed to compute the orthologous substitution rates used in the previous regressions, but the resulting number of aligned CpG bases in human-macaque is too small for meaningful substitution rate estimation. Therefore, we now remove orthologous substitution rates from the predictor list. Results concerning other predictors remain largely unchanged for the all sites and non-CpG sites regressions (data not shown).

In contrast, neutral rates computed from CpG sites present a different behavior: the regression explains a substantially larger share of variability (R^2 ^= 82%), and only three predictors are significant; namely GC content, exon density, and autosome/X indicator (Table 3). Differences in the sets of significant predictors for non-CpG and CpG rates are consistent with the hypermutability and different molecular mechanisms affecting the evolution of CpG sites [34]. Most substitutions here are deaminations from CpG to TpG or CpA sites, which occur at a higher rate when cytosine is methylated.

**Table 3:** Regression results for neutral substitution rates estimated from CpG sites

	CpG sites			
	
Predictors	*t *value*	Significance^†^	VIF^‡^	Variability explained^§^
X chromosome/autosome indicator	13.99	<10^-4^	1.1	0.02
GC content				
Linear term	-57.37	<10^-4^	2.7	0.32
Quadratic term	5.73	<10^-4^	1.2	
Exon density	-6.28	<10^-4^	2.3	0.003
SNP density	NS	NS	NS	NS
Male recomb rate	NS	NS	NS	NS
Female recomb rate	NS	NS	NS	NS
Distance to telomeres				
Linear term	NS	NS	NS	NS
Quadratic term	NS	NS	NS	
Multiple R^2^				0.82
Adjusted R^2^				0.82

CpG sites were taken in ancestral repeats (without requiring orthology to mouse, rat, dog and cow) for each of 2,270 windows of size 1 Mb. *t value, test statistic of null hypothesis that each predictor's coefficient is equal to zero; ^†^*p*-values adjusted for multiple tests (using Bonferroni correction); ^‡^VIF, variance inflation factor; ^§^relative contribution to explained variability computed for each predictor. NS, non-significant.

**Figure 2:**
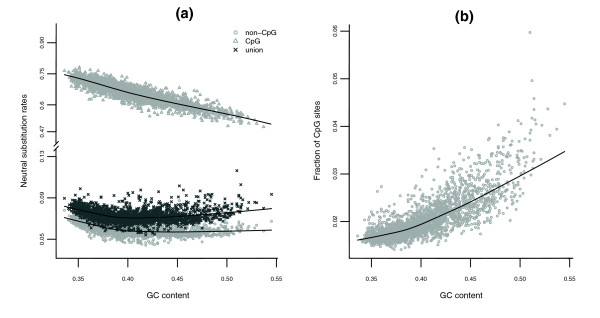
Neutral rates, GC and CpG content. Scatter plots of **(a) **human-macaque JC neutral substitution rates against GC content, for CpG sites (triangles), non-CpG sites (circles), and 'union' sites (crosses), and **(b) **fraction of CpG sites against GC content. Each point represents one of 2,270 windows of size 1 Mb. Lowess smoothers are superimposed to the plots to help visualize the relationships. Note the different scales on the truncated y-axis for (a).

Remarkably, the three significant predictors for CpG rates are known to be associated with methylation patterns. It has been reported that unmethylated sequences tend to concentrate in high-GC and gene-rich regions of the genome [35]. GC content is an even more prominent predictor for the CpG substitution rate (variability explained 32%) than for the non-CpG and all sites rates. Moreover, the curvature is much less pronounced (albeit still significant), and thus the negative correlation is more clear-cut (r = -0.88; Figure 2a). Exon density also has a strong negative association with the CpG rate (r = -0.65) and is a highly significant negative predictor in the regression (although its variability explained is negligible due to its correlation with GC content, r = 0.7). The marked negative associations between CpG rates and both GC content and exon density suggest that substitutions at CpG sites are indeed less frequent in regions with lower methylation levels for the CpG dinucleotides. The autosome/X indicator has a highly significant positive effect on CpG rates, but is less of an outstanding predictor than for non-CpG and all sites rates (variability explained 2%). This is consistent with previously reported evidence for weaker male mutation bias at CpG sites [29].

A few technical remarks are in order before moving to further analyses: correlations among the genomic features we used as predictors are not strong enough to jeopardize the quality of our regression fits or our ability to quantify individual predictive contributions (variance inflation factors are all small to moderate, and always below 10; Tables 1 and 3, and Materials and methods). However, these correlations are strong enough to complicate the interpretation of some regression output; for instance, they may account for the relatively low variability explained measurements for male recombination rate and exon density, despite their high significance in our models. Moreover, since genomic features do indeed have substantial and complex relationships with one another, we must remain aware of the possibility that some of the predictors included in our regressions may act as 'proxies' for other features, which affect substitution rate variation but are not included in the models.

### Relationship between neutral rates and GC content

Next, we investigate in more detail the non-linear, biphasic relationship between neutral substitution rates and GC content by considering human-macaque substitution rates computed from: non-CpG sites; CpG sites, defined as CG in either the human or the macaque sequence; and the union of these two categories. On average, this union leaves out about a third of all sites in ancestral interspersed repeats as sites that cannot be confidently classified as either CpG or non-CpG (see Materials and methods for details).

The relationship between substitution rates and GC content for 'union' sites presents a pronounced curvature, with both a descending branch at low GC content levels and an ascending branch at high GC content levels. For non-CpG sites, a curvature also exists, but the ascending branch is much weaker. For CpG sites, however, the picture is quite different; substitution rates at these hypermutable sites are much higher, and they present a negative, nearly linear relationship with GC content (Figure 2a) - these observations are consistent with the decreasing magnitude of the quadratic coefficient for GC content when passing from all sites, to non-CpG sites, to CpG sites in the regressions presented above (see *t*-values in Tables 1 and 3).

In the Materials and methods section we provide some theoretical derivations showing that the rates from 'union' sites behave as a convex linear combination of the rates from non-CpG and CpG sites, with weights given by the fractions of the two types of sites. Since the fraction of CpG sites increases markedly with GC content (Figure 2b), the ascending branch presented by rates from 'union' sites at high GC levels can be explained as a consequence of the increasing dominance of hypermutable CpG sites in the convex combination. Interestingly, the data show a non-linear increase for the fraction of CpG sites - a pattern that is consistent with the expectation for such a fraction derived under a simple assumption of site independence. Hellmann *et al*. [8] suggested that a curved relationship between substitution rates and GC content may be due to an underlying quadratic relationship between the probability of observing a CpG site and GC content itself. According to our derivations, this increase need not be quadratic to explain the biphasic nature of the relationship between substitution rates and GC content; however, our data do support a quadratic increase. Interestingly, high mutation rates at CpG sites were found to reduce the silent substitution rate (K_S_) in GC-poor regions and to increase it in GC-rich regions [36].

### Neutral rates and prediction of functional elements

Predicting the location of functional sequences in the human genome is a very important and active research area (recent examples include [37-39]). It was also noted that predictions generated by several methods are negatively correlated with neutral substitution rates in ENCODE regions [40]. Here we investigate in more detail the relationship between neutral substitution rates and the genome-wide distributions of several classes of predicted and experimentally mapped functional elements. A list of these classes, with short descriptions and references, is given in Table 4 (first four columns).

**Table 4:** Associations between human-macaque neutral substitution rates and frequencies of various classes of functional elements

Class of elements	Short description	Conservation based	Reference	Correlation coefficient	Partial correlation coefficient
phyloHMM (P)	Predicted functional elements; highly conserved non-exonic sequences identified by phyloHMM	Yes (17 vertebrate species)	[52]	-0.32	-0.38
ESPERR-RP (P)	Predicted regulatory elements; non-exonic sequences with high regulatory potential, as measured by the ESPERR-RP score	Yes (7 mammalian species)	[39]	-0.24	-0.30
Enhancers (P)	Predicted enhancers; non-exonic sequences under strong constraint in human-rodent comparisons	Yes (human, mouse, rat)	[38,48]	-0.06	-0.22
CTCF-binding sites (P)	Predicted CTCF binding sites; identified by single sequence motif finding methods	No	[41]	-0.12	-0.10
CTCF-binding sites (E)	Experimentally mapped CTCF binding sites	No	[41]	-0.20	-0.08
ER binding sites (E)	Experimentally mapped estrogen receptor binding sites	No	[42]	-0.14	-0.09
RNA polymerase II binding sites (E)	Experimentally mapped RNA polymerase II binding sites	No	[42]	-0.11	0.01

Pearson's correlation and partial correlation coefficients. The substitution rates are estimated from all sites in ancestral repeats (without requiring orthology to mouse, rat, dog and cow) for each of 2,270 windows of size1 Mb.

Computing frequencies for each class in our 1 Mb windows, and correlating these with neutral substitution rates (Table 4, fifth column), we observe strong negative associations for predictions based on conservation (except for predicted enhancers) and sizeable but generally weaker negative associations for predictions that do not rely on conservation and experimentally mapped elements. Since frequencies of both predicted and experimentally mapped functional elements also correlate with the genomic features used in our regressions for neutral rates (data not shown), the question is whether the correlations with neutral rates are merely a byproduct of the correlations with GC content, gene density, and so on.

To address this question, we compute the partial correlations between frequencies and neutral rates, given all the predictors used in our regression models except for the orthologous mouse-rat and dog-cow rates (Table 4, sixth column). These partial correlations are correlations between the residuals from regressing each of the frequencies and the neutral rates on our set of genomic features. If the associations between neutral rates and frequencies of predicted and/or experimentally mapped regulatory elements were due only to associations with these features, the partial correlation coefficients should be much closer to zero than the original correlation coefficients. For predictions that do not rely on conservation and for experimentally mapped elements, partial correlations indeed decrease in size compared to the original correlations. These decreases are substantial (except in the case of predicted binding sites of the transcription factor CTCF), showing that the original correlations can be at least partially explained by strong correlations between frequencies of the binding sites and, say, gene density [41,42]. In contrast, for predictions based on conservation (including predicted enhancers), partial correlations are in fact stronger than the original ones. Thus, accounting for co-variation with the genomic features considered in our study does not 'explain away' the negative association between neutral rates and frequencies of conservation-based predicted functional elements conservation, but rather it allows this association to emerge more clearly.

These results indicate that accounting for local neutral rates can improve predictions of functional elements in the genome, particularly when conservation-based methods are employed. As a preliminary evaluation, we compared the sensitivity of ESPERR-RP scores (evolutionary and sequence pattern extraction through reduced representations-regulatory potential scores) for identifying experimentally mapped elements, with and without a simple neutral rate correction that increases the score at locations evolving faster than expected on the basis of their genomic features (see Materials and methods). The relative change in the fraction of experimentally mapped elements that are intersected by ESPERR-RP predictions, before and after correction, is 0.23 for the estrogen receptor class, 0.07 for RNA polymerase II, and 0.31 for CTCF. Thus, consistent with the nature of the correction, sensitivity can increase substantially in fast-evolving regions. However, the magnitude of the increase varies broadly among the three classes, and is accompanied by a relative change of 0.18 in the overall number of predictions; this is likely to produce some loss in specificity, although this possibility cannot be assessed directly without reference to a negative set.

## Conclusion

In this study, we examine regional variation in neutral substitution rates along the human genome utilizing its alignments with the macaque sequence. Analysis of human-macaque rates is of crucial importance because this evolutionary distance produces divergence estimates that are likely to be much more accurate than those used in previous studies.

We used multiple regression techniques to investigate a number of features as predictors of variation in neutral rates, including variables already considered in the literature (for example, GC content, exon density, SNP density), variables whose definition we modified as to be able to detect subtler associations (for example, separate male and female recombination rates, distance to telomeres considering positions in both human and macaque), and novel variables (for example, location on chromosome X versus autosomes, neutral substitution rates computed from orthologous regions in pair-wise alignments of mouse with rat, and dog with cow). Although the correlations among these predictors and the lack of data on other potentially relevant features complicate some aspects of the analysis, we are able to provide an effective characterization of the association between multiple genomic features and neutral substitution rates.

Our regressions explain approximately 52% of the variation in human-macaque substitution rates calculated from all and non-CpG bases in ancestral repeats, and 82% for rates calculated from CpG bases. They confirm previously reported associations, reveal new ones, and support the notion of substantially different processes underlying mutations at CpG and non-CpG sites.

The regressions confirm a biphasic relationship between neutral substitution rates and GC content [3,8]. We also provide insights on the determinants of its curvature with a separate analysis of neutral rates computed at CpG, non-CpG, and the 'union' of CpG and non-CpG sites. Our data indicate that, as GC increases: substitution rates for CpG sites decrease almost linearly (possibly due to reduced methylation of sites in CpG islands at higher GC levels); substitution rates for non-CpG sites have a dominant decreasing trend, with a modest increase at higher GC levels; and substitution rates for union sites present both a descending branch at low GC levels and an ascending branch at high GC levels. With some mathematical derivations, we show that this ascent, and hence the pronounced curvature in the relationship between neutral rates computed from union sites and GC content, can be due to an increase in the fraction of faster evolving positions, that is, CpG sites. As for the more modest ascent observed for substitution rates at non-CpG sites, a possible cause could be a higher mutational propensity of C and G bases (compared to A and T), even outside of CpG dinucleotides [43,44]. The dominant negative trend for non-CpG sites, where most substitutions are replication-based, could be associated with replication timing. Regions with high GC content are known to replicate earlier [45] and might be less prone to replication errors and/or be repaired more efficiently, than late replicating AT-rich DNA. In turn, the negative trend observed for replication independent CpG deaminations can be explained by lower methylation levels in high GC regions.

Our regressions also identify male (as opposed to female) recombination and autosomal versus non-autosomal location as significant predictors of divergence. The role of recombination has been investigated in other studies [2,3,8], and our own results must be interpreted as preliminary, since the resolution we employ (1 Mb windows) may be too low to capture some important effects of variation in rates of recombination, which is believed to occur at a smaller scale [25]. However, consistent with the hypothesis that female recombination affects GC content [24], we find that separating male and female rates is crucial to detect recombination as a mutagenic mechanism, at least at a 1 Mb resolution.

Finally, our regressions strongly suggest the existence of yet unidentified mutagenic mechanisms, whose effects are shared across mammalian genomes and are quite substantial compared to the mechanisms captured by the other variables we considered. Some of these mechanisms might concern regional differences in repair, proximity to origins of replication, density of matrix attachment sites, and so on. We note that our analysis excludes regions of the human genome that have diverged so much that orthologs cannot be reliably assigned in mouse, rat, dog and cow. Therefore, some caution should be exercised in extrapolating the outcomes of our regressions to such regions.

As more data become available, incorporating additional predictors in the regressions may be beneficial. Of special interest would be data on other species. A rigorous statistical comparison of mutagenic mechanisms across different genomes would require computing the same set of predictors for all genomes under consideration, something that is not currently achievable. For example, a recombination map for the macaque genome would allow us to elucidate the effect of proximity to telomeres (if distance to telomeres as defined in this study merely proxies macaque recombination, including the latter in a regression should dramatically deplete the significance of the former).

The strong negative correlations we observe between neutral rates and frequencies of predicted functional elements based on conservation suggest that these predictions tend to concentrate in slowly evolving regions of the genome, resulting in a lack of sensitivity in fast evolving regions. In comparison, the negative correlations between neutral rates and frequencies of experimentally mapped elements are weaker, and at least partially explained by co-variation with other genomic features. Our preliminary calculations confirm that even a very simple correction can improve sensitivity in regions of the genome that evolve faster than expected given their genomic features. However, the improvement varies substantially among different classes of experimentally mapped elements, and involves a potential loss in specificity. A more in-depth investigation of this topic will require analyzing more sophisticated correction mechanisms, and ways to combine corrections with the segmentation algorithms producing prediction intervals. It is also possible that the scale at which neutral rate variation is most usefully incorporated for functional element prediction may be smaller than the 1 Mb used here, and that considering additional classes of validated elements would clarify results.

## Materials and methods

### Data preparation

We used 1 Mb non-overlapping windows to cover all autosomes and chromosome X from the latest release of the human genome, hg18. This window size was found to be informative and effective in studies of substitution rates in rodents (for example, [12]). Moreover, it allowed us to include in our regressions sex-specific recombination rates [23], which are not available at smaller scales. For each such window, using annotations and tracks at the UCSC Human Genome Browser [20], we computed GC content, exon density, SNP density (based on dbSNP126; we opted not to use Hapmap data because of its heavy bias against recent repeats), and sex-specific recombination rates from deCODE [23]. For each window, we also defined an indicator variable, equal to 1 for windows in autosomes and pseudo-autosomal portions of chromosome X, and 0 for windows in the non-pseudo-autosomal X.

Using 17-way MultiZ alignments [46] at the UCSC Genome Browser, we retrieved pair-wise alignments of repeats annotated by the Repeat Masker that are at least 60% alignable between human and each of macaque, dog, cow, mouse and rat, excluding the following families: Alu, simple repeats, low complexity regions, RNA and satellite repeats. We also excluded repeats that are located in regions of either human or macaque segmental duplications, as annotated in the UCSC Genome Browser, since duplicated regions might not be true orthologs.

We defined non-CpG sites for pairwise alignments as those that are not CG in both species, and not immediately preceded by C or followed by G in either species. Using simulation experiments, Meunier and Duret [24] showed that this definition of non-CpG sites effectively captures sites that evolved without being parts of CpGs at the human-chimpanzee distance. The same definition was successfully used in the study by Gaffney and Keightley [12] for the mouse-rat distance. CpG sites were defined as sites for which C was immediately followed by G (or G immediately preceded by C) at least in one of the species.

Mapping of the selected repeats and other data retrieved from the Genome Browser onto 1 Mb windows and miscellaneous data formatting procedures were performed using Galaxy [47]. Since the number of repeat bases used in the substitution rates calculation differed greatly from window to window, we filtered out windows where the number of informative non-CpG columns in any of the pair-wise alignments was less then 5K (resulting in 2,270 windows). The selected windows provide a fairly uniform coverage of the human genome. Substitution rates were calculated using both the JC [18] (results reported throughout the paper) and the HKY [19] (Additional data file 1) models. For CpG sites we calculated rates for each of the windows selected in the previous step, but without requiring that repeats be 60% alignable between human and each of macaque, dog, cow, mouse and rat.

The sets of predicted and experimentally assessed functional elements were retrieved from various online sources. The highly conserved elements produced by phyloHMM were retrieved from the UCSC genome browser 'most conserved' track [17], with regions overlapping known exons filtered out. Predicted enhancers were obtained from a set available at the VISTA enhancers browser (see links provided in [38,48]) - this is a set of human non-coding sequences obtained thresholding a constraint score from human-mouse-rat comparisons. Computationally predicted and experimentally assessed CTCF binding sites were downloaded from the website provided in [41], and experimentally assessed estrogen receptor and RNA polymerase II binding sites were obtained from the website provided in [42]. When necessary, downloaded coordinates were lifted over to hg18. Elements having high ESPERR regulatory potential [39] were defined as stretches of sequence having an ESPERR-RP score of at least 0.05 for at least 200 bp, and not overlapping exons in the known genes set [49].

The correction for the ESPERR-RP score is defined as:

$$RP*(b)=RP(b)+\text{max}\left(\text{0,}\frac{r_{w(b)}-{\hat{r}}_{w(b)}}{{\hat{r}}_{w(b)}}\right)$$

where *b *is a base, *w*(*b*) is the 1 Mb window to which the base belongs, and *r *and $\hat{r}$ represent, respectively, the observed neutral rate and the fitted value from our regression model. Elements with high corrected ESPERR-RP are then defined using exactly the same segmentation rule applied to uncorrected scores. Validated elements in a given class that are intersected by predictions (from original or corrected scores) are defined as elements that have at least one prediction interval overlapping them by 50 bp or more.

### Regression analysis

All regressions were implemented using ordinary least squares; the rates for our set of 2,270 windows did not present strong auto-correlations, and using a generalized least squares fit to take into account response auto-correlations gave very similar results. Also, notwithstanding the sizeable correlations among predictor variables, the least square fits were not unduly affected by multi-collinearity. To assess the degree by which multi-collinearity among the predictors influenced stability and accuracy of regression estimates, we calculated the variance inflation factors (VIF) [50] for all predictors in each of our models.

To evaluate predictors in a regression model, we calculated the relative contribution to the explained variability for each individual predictor, given all other predictors in the model, as the relative increase in the determination coefficient R^2 ^(overall share of explained variability) due to including that predictor:

$$RCVE=\frac{R_{full}^{\text{2}}-R_{reduced}^{\text{2}}}{R_{full}^{\text{2}}}$$

where $R_{full}^{\text{2}}$ is the R^2 ^of the full model (with all predictors), while $R_{reduced}^{\text{2}}$ is the R^2 ^for the model obtained from the full model dropping the predictor of interest. The relative contributions to variability explained (RCVEs) are similar to partial correlations [50], and in regressions with correlated predictors they must be interpreted in context because they do not represent a partition of $R_{full}^{\text{2}}$. Nevertheless, they allow us to quantify the explanatory contribution of each individual predictor beyond its associations with other predictors (when predictors are correlated, the R^2^s from each univariate regression are not a meaningful measurement of their contributions).

### Theoretical derivations for the analysis of substitution rates versus GC content

A JC substitution rate, say *d*^*JC*^(*p*), is a convex, monotone increasing function of *p*, the proportion of mismatches among the positions considered in its calculation:

$$d^{JC}(p)=-\frac{3}{4}\ln\left(1-\frac{4}{3}p\right)$$

Moreover, when considering a collection of positions (for example, positions in ancestral repeats within a given window) comprising both CpG and non-CpG sites, the overall proportion of mismatches, say *p*_*all*_, can be decomposed as a weighted average of the proportions of mismatches at CpG and non-CpG sites:

*P*_*all *_= *f*_*CpG*_·*p*_*CpG *_+ (1 - *f*_*CpG*_)·*p*_*non*-*CpG*_,

where the weight *f*_*CpG *_is the general proportion of CpG sites (matching and mismatching). Since hyper-mutability of CpG sites implies that *p*_*CpG *_is higher than *p*_*non*-*CpG*_, and because of the monotonicity and convexity of *d*^*JC*^(.), we have:

*d*^*JC *^(*p*_*non*-*CpG*_) <*d*^*JC *^(*p*_*all*_) ≤ *f*_*CpG*_·*d*^*JC *^(*p*_*CpG*_) + (1 - *f*_*CpG*_)·*d*^*JC *^(*p*_*non*-*CpG*_) <*d*^*JC*^(*p*_*CpG*_)

It follows that the rate computed at all sites will take a value intermediate between that of the rate computed at non-CpG sites and that of the rate computed at CpG sites, with a possibility to be closer to the latter the higher the proportion of CpG sites, *f*_*CpG*_. The fact that *f*_*CpG *_grows with GC content (Figure 2b) therefore allows *d*^*JC*^(*p*_*all*_) to be 'pulled towards' *d*^*JC*^(*p*_*CpG*_) at high GC levels, and can explain the ascending branch at high GC presented by substitution rates calculated on all sites (Figure 2a). Although we conducted both data analysis and theoretical derivations in terms of JC rates, a similar rationale should extend to rates based on more complicated models, such as HKY [19] or REV (time reversible substitution model) [51].

## Abbreviations

ESPERR-RP, evolutionary and sequence pattern extraction through reduced representations-regulatory potential score; HKY, Hasegawa-Kishino-Yano; JC, Jukes-Cantor; SNP, single nucleotide polymorphism; VIF, variance inflation factor.

## Additional data files

The following additional data are available with the online version of this paper: Additional data file 1 is a table that lists results of the regression analyses for the neutral substitution rates estimated using the HKY model.

## Supplementary Material

Additional data file 1 Results of the regression analyses for the neutral substitution rates estimated using the HKY model. Click here for file
