# Supplementary material for: Human-macaque comparisons illuminate variation in neutral substitution rates
Source: Genome Biol. 2008 Apr 30;9(4):R76. doi: 10.1186/gb-2008-9-4-r76 (PMC2643947; doi:10.1186/gb-2008-9-4-r76)
Supplement: Additional data file 1 — Results of the regression analyses for the neutral substitution rates estimated using the HKY model. [file gb-2008-9-4-r76-S1.pdf]

<u>Supplementary Table S1</u>: Regression results for neutral substitution rates estimated from non-CpG, all, and CpG sites using the HKY85 model.

Predictors	Non-CpG sites				All sites				CpG sites			
	t value[a]	Signif [b]	VIF[c]	Variability explained[d]	*t* value[a]	Signif [b]	VIF[c]	Variability explained[d]	*t* value[a]	Signif [b]	VIF[c]	Variability explained[d]
X chromosome/ autosome indicator	13.05	$<10^{-4}$	1.2	0.07	14.34	$<10^{-4}$	1.3	0.08	8.56	$<10^{-4}$	1.1	0.01
GC content (linear term) / *GC content (quadratic term)*	-9.69	$<10^{-4}$	3.7	0.11	-4.61	$<10^{-4}$	3.3	0.14	-40.39	$<10^{-4}$	2.7	0.25
Exon density	-6.75	$<10^{-4}$	2.4	0.02	-9.07	$<10^{-4}$	2.4	0.03	-12.92	$<10^{-4}$	2.3	0.02
SNP density	6.06	$<10^{-4}$	1.2	0.02	6.6	$<10^{-4}$	1.2	0.02	n.s.[e]	n.s.	n.s.	n.s.
Male recomb rate	3.52	0.005	1.6	0.01	4.29	$<10^{-4}$	1.6	0.01	3.97	$<10^{-4}$	1.6	0.002
Female recomb rate	n.s	n.s.	n.s	n.s	n.s	n.s	n.s	n.s	n.s.	n.s.	n.s.	n.s.
Distance to telomere (linear term)	-12.22	$<10^{-4}$	2.5		-16.61	$<10^{-4}$	2.5		n.s.	n.s.	n.s.	
Distance to telomere (quadratic term)	7.64	$<10^{-4}$	2	0.06	10.75	$<10^{-4}$	2	0.11	n.s.	n.s.	n.s.	n.s.
Mouse-rat orthologous neutral rate	8.75	$<10^{-4}$	1.8		7.52	$<10^{-4}$	1.4		NA	NA	NA	
Dog-cow orthologous neutral rate	10.42	$<10^{-4}$	1.4	0.09	10.31	$<10^{-4}$	1.4	0.07	NA	NA	NA	NA
Multiple R^2				0.51				0.52				0.76
Adjusted R^2				0.51				0.52				0.76
Number of windows used[f]				2263				2263				2247

[a]*t* value – test statistic of null hypothesis that each predictor's coefficient is equal to zero; [b]*P* values adjusted for multiple tests (using Bonferroni correction); [c]VIF – variance inflation factor; [d]Relative contribution to explained variability computed for each predictor; [e]n.s. – non-significant.

[f] while JC rates were estimated on 2270 windows, HKY85 rates could be computed on most (but not all) such windows. We used the phyloFit software (A. Siepel and D. Haussler. 2004. Phylogenetic estimation of context-dependent substitution rates by maximum likelihood. Mol. Biol. Evol., 21:468-488.).
